# Repetitive transcranial magnetic stimulation induces oscillatory power changes in chronic tinnitus

**DOI:** 10.3389/fncel.2015.00421

**Published:** 2015-10-21

**Authors:** Martin Schecklmann, Astrid Lehner, Judith Gollmitzer, Eldrid Schmidt, Winfried Schlee, Berthold Langguth

**Affiliations:** Department of Psychiatry and Psychotherapy, Interdisciplinary Tinnitus Clinic, University of RegensburgRegensburg, Germany

**Keywords:** ringing in the ears, TMS, EEG, plasticity, frontal cortex, temporal cortex

## Abstract

Chronic tinnitus is associated with neuroplastic changes in auditory and non-auditory cortical areas. About 10 years ago, repetitive transcranial magnetic stimulation (rTMS) of auditory and prefrontal cortex was introduced as potential treatment for tinnitus. The resulting changes in tinnitus loudness are interpreted in the context of rTMS induced activity changes (neuroplasticity). Here, we investigate the effect of single rTMS sessions on oscillatory power to probe the capacity of rTMS to interfere with tinnitus-specific cortical plasticity. We measured 20 patients with bilateral chronic tinnitus and 20 healthy controls comparable for age, sex, handedness, and hearing level with a 63-channel electroencephalography (EEG) system. Educational level, intelligence, depressivity and hyperacusis were controlled for by analysis of covariance. Different rTMS protocols were tested: Left and right temporal and left and right prefrontal cortices were each stimulated with 200 pulses at 1 Hz and with an intensity of 60% stimulator output. Stimulation of central parietal cortex with 6-fold reduced intensity (inverted passive-cooled coil) served as sham condition. Before and after each rTMS protocol 5 min of resting state EEG were recorded. The order of rTMS protocols was randomized over two sessions with 1 week interval in between. Analyses on electrode level showed that people with and without tinnitus differed in their response to left temporal and right frontal stimulation. In tinnitus patients left temporal rTMS decreased frontal theta and delta and increased beta2 power, whereas right frontal rTMS decreased right temporal beta3 and gamma power. No changes or increases were observed in the control group. Only non-systematic changes in tinnitus loudness were induced by single sessions of rTMS. This is the first study to show tinnitus-related alterations of neuroplasticity that were specific to stimulation site and oscillatory frequency. The observed effects can be interpreted within the thalamocortical dysrhythmia model assuming that slow waves represent processes of deafferentiation and that high frequencies might be indicators for tinnitus loudness. Moreover our findings confirm the role of the left temporal and the right frontal areas as relevant hubs in tinnitus related neuronal network. Our results underscore the value of combined TMS-EEG measurements for investigating disease related changes in neuroplasticity.

## Introduction

Tinnitus is the perception of sound in the absence of a corresponding external auditory stimulus. Chronic tinnitus is experienced in about 5–15% of the general population and severely impairs the quality of life in about 1–2% (Axelsson and Ringdahl, 1989; Khedr et al., 2010b; Shargorodsky et al., 2010). Abnormal auditory input—e.g., by cochlear damage—is considered a frequent trigger, but not a sufficient condition to develop chronic tinnitus (Moller, 2007). Decreased output from the cochlea leads to neuroplastic changes along the auditory pathway including changes of the spontaneous firing rate, evoked activity and tonotopic reorganization. In addition to alterations in the auditory pathways, neuroplastic changes have also been detected in non-auditory brain areas (for an overview; De Ridder et al., 2011, 2014).

But although increasing knowledge about tinnitus generation has been revealed by neuroscientific research during the last decades, the pathophysiology of tinnitus is still incompletely understood and there exists no well established causally oriented treatment (Langguth et al., 2013). For the identification of the neuronal correlates of tinnitus functional (fMRI) and structural magnetic resonance imaging (MRI), positron emission tomography (PET), single photon emission computed tomography (SPECT) as well as electro- and magneto-encephalography (EEG, MEG) have been used (for an overview Adjamian et al., 2009; Lanting et al., 2009). Studies using these methods have already added important information to tinnitus research. Nevertheless we have to be aware that each of these methods has both strengths and weaknesses. As tinnitus is continuously perceived in most cases, resting state measurements were assumed to be best suited to identify the neuronal correlates of tinnitus. However, in most of the EEG-, MEG-, PET- and fMRI-resting state studies perceptual, attentional and cognitive processes during the measurement were not specified. Moreover variations in tinnitus perception during measurement were not assessed. Resting state studies assume that the tinnitus percept and the related neural changes are constant over time. In a recent analysis a substantial variability of oscillatory brain activity during the measurements was reported (Schlee et al., 2014). An alternative approach to resting state is to measure, whether the brain’s reaction to an external stimulus differs in tinnitus patients as compared to healthy controls. Most such studies investigated sound-evoked activity. Interpretation of these data is difficult as associated co-morbidities such as hearing loss and hyperacusis have to be considered (Gu et al., 2010; Melcher et al., 2013; Schecklmann et al., 2013). If a tinnitus-like sound is used for stimulation it is a challenge to exactly measure the pitch and volume of the tinnitus (Hoare et al., 2014). Furthermore, sound-evoked activity is always associated with co-activation of auditory and non-auditory (e.g., attention network) areas thus hampering the focused examination of single areas. Other studies contrasted conditions where patients were instructed to distract or to focus on their tinnitus by using cognitive tasks (Andersson et al., 2006). Results obtained with such techniques are confounded by activation related to the cognitive effort. Similar problem holds true for other tinnitus suppression methods such as lidocaine injection or somatic manoeuvres (e.g., eye movements). Moreover these techniques are limited by invasiveness or low prevalence of such kind of patients, respectively (Lanting et al., 2009).

Due to these limitations, it is important to collect and combine information from different methods in order to obtain an elaborate knowledge about the neuronal correlates of chronic tinnitus. Here, we suggest the use of another method which might overcome some of the above mentioned limitations: combined measurements of transcranial magnetic stimulation (TMS) and EEG to investigate differences between people with and without tinnitus in the reaction to magnetic stimulation. The rationale of this approach is that the way how the brain reacts to a specific stimulation protocol differs between people with and without tinnitus. The information about tinnitus-related brain reagibility may in turn provide a deeper understanding of tinnitus pathophysiology (Langguth et al., 2012; Müller et al., 2013). TMS-EEG studies enable the measurement of neural reactivity (TMS evoked activity after single pulses) and of neuroplasticity (resting state measurements before and after short repetitive transcranial magnetic stimulation (rTMS) interventions) free of cognitive confounders. Single pulses induce immediate neuronal responses which can be measured by concomitant EEG (Rogasch et al., 2013; Van Doren et al., 2015). Repetitive TMS (rTMS; rhythmic repetition of single pulses at specific frequencies or with specific protocols) induces prolonged changes for up to 2 h which can be measured by resting state EEG before and after the intervention (Thut and Pascual-Leone, 2010). Combined TMS-EEG measurements have been shown to detect disease-related alterations of neuroplasticity as indicated for example in schizophrenia research (McClintock et al., 2011; Rogasch et al., 2014). To sum up, combined TMS-EEG studies enable the measurement of TMS evoked activity after single pulses (neural reactivity) and of changes in resting state activity from before to after a single sessions of rTMS (neuroplasticity). For the present study we used TMS-EEG to investigate alterations of neuroplasticity in tinnitus.

Several studies have shown a transient reduction of tinnitus loudness after single sessions of rTMS (Plewnia et al., 2003; De Ridder et al., 2005). Repeated daily sessions of rTMS over temporal and frontal areas have been investigated as therapeutic tool in chronic tinnitus (Kleinjung et al., 2008; Kreuzer et al., 2011; Lehner et al., 2013; Langguth et al., 2014). However, treatment effects are only small and individually highly variable (Langguth et al., 2012). In most studies low-frequency stimulation of the temporal cortex was investigated albeit other frequencies and areas have also been stimulated so far (Khedr et al., 2008; Lehner et al., 2013). The rational of this approach is to reduce putative over-activation and hyper-connectivity in chronic tinnitus (Eichhammer et al., 2003).

Here, we investigated the effects of single sessions of low-frequency rTMS over the left and right temporal and prefrontal cortices on oscillatory brain activity. We concentrated on effects of 1 Hz rTMS as there is most clinical evidence for 1 Hz stimulation in chronic tinnitus. Furthermore, a direct comparison with other protocols revealed that 1 Hz rTMS produces the most reliable changes in oscillatory brain activity (Müller et al., 2013). We aimed at testing if this procedure can be used (1) as marker for altered neuroplasticity in chronic tinnitus (pre- to post-rTMS effects); and (2) as indicator for rTMS induced tinnitus loudness reduction. Concerning the first question we hypothesized that neuroplastic effects of rTMS over temporal and frontal brain areas will differ between tinnitus patients and controls. Numerous TMS-EEG-studies in healthy subjects have investigated effects of different rTMS protocols on different EEG measures (evoked potentials, TMS-evoked potentials, resting state power) (for review, see Thut and Pascual-Leone, 2010; Chung et al., 2015). 1 Hz stimulation of prefrontal and temporal cortex in healthy controls showed heterogeneous findings with respect to changes in oscillatory power (Schutter et al., 2001; Kim et al., 2012; Woźniewska et al., 2013). One study in chronic tinnitus showed marginally reduced tinnitus loudness and gamma band power in the stimulated auditory cortex (Müller et al., 2013). Gamma is indicated as neural marker for tinnitus loudness (Vanneste and De Ridder, 2012). Based on these findings together with numerous findings of increased temporal and prefrontal activity and connectivity in chronic tinnitus (for review, see De Ridder et al., 2011, 2014), we deduced the hypothesis that tinnitus loudness reductions are associated with reductions in gamma power. Moreover, we expected that the increased connectivity of frontal and auditory areas in tinnitus will result in remote rTMS effects in the tinnitus group.

## Materials and Methods

### Subjects and Study Procedures

Patients with chronic bilateral tinnitus, who had consulted the multidisciplinary Tinnitus Clinic of the University of Regensburg, were included in the study. Subjects with history or presence of severe and relevant somatic, neurologic, or mental disorders were not included. Intake of psychotropic medication, participating in rTMS interventions within 1 year before the present study and wearing metal implants were further exclusion criteria. The control group was recruited by advertisements and matched for age, sex, handedness, education, and hearing level. The study was approved by the Ethics Committee of the University of Regensburg (12-101-0216). All participants gave written informed consent after a comprehensive explanation of the procedures.

After signing the consent form all participants completed the tinnitus questionnaire (Hallam et al., 1988; Goebel and Hiller, 1994), the Major Depression Inventory (Bech et al., 2001), the German questionnaire for hyperacusis (GÜF; Nelting et al., 2002), a numeric rating scale with respect to tinnitus loudness, the MWT-B—a measure for verbal intelligence (Lehrl, 2005) and the ZVT—a measure for general processing speed free from language performance (Oswald and Roth, 1987). In addition participants answered questions with respect to demographic and tinnitus-related characteristics, usage of psychoactive substances, and handedness (Oldfield, 1971). Furthermore, hearing level was measured with a standard audiogram using frequencies ranging from 125 Hz to 8 kHz (Madsen Midimate 622D; GN Otometrics, Denmark).

**Figure 1:**
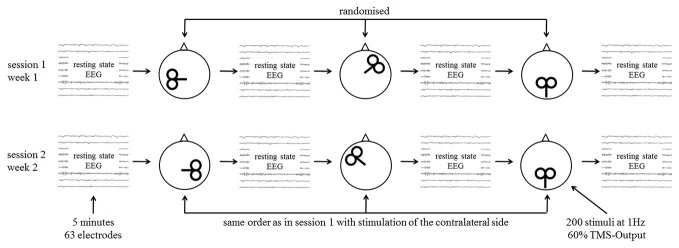
**Study procedures with single sessions of repetitive transcranial magnetic stimulation (rTMS) and measurement with resting state electroencephalography (EEG)**.

With an interval of 2 weeks, two TMS-EEG sessions were performed (Figure 1). Measurements were done on the same weekday and at the same time except in two cases in the patient and in two cases in the control group. At each session three rTMS protocols were tested and four EEG recordings (before and after each rTMS intervention) were done. The TMS motor threshold was assessed with the EEG cap on the head at the beginning of each session. EEG and rTMS measurements were done with insert ear-plugs.

The order of the different stimulation protocols was randomized. Randomization was guided by the rationale to stimulate one frontal, one temporal, and one parietal (=sham) cortical site per session (Figure 1), resulting in 24 possible combinations for the first session. In the second session the contralateral stimulation (sham stimulation was unchanged for both measurements) sites were stimulated in the same order as in the first session. The 24 possible combinations were randomized before study begin for the patient and control group separately.

Twenty four patients with chronic tinnitus were included in the study, but only the data of 20 patients could be analyzed. Three patients did not complete both sessions and data of one patient could not be analyzed because of a high amount of artifacts in the EEG recordings. These four patients had been randomized, but were excluded from the analysis. We measured 20 healthy controls which were comparable to the patients for age, sex, handedness, education, and hearing level. Thus, the last four randomization orders were not measured. In all participants, educational years, hyperacusis, depressivity and intelligence were recorded and considered in the statistical analysis. To control for group differences we used analyses of covariance (see “Data Analysis” Section). For sample-, tinnitus-, and measurement-related details see Table 1.

**Table 1:** **Demographic, tinnitus-related, and measurement-related data of the patient and control group**.

	Tinnitus patients	Healthy controls	Statistics
Age (years)	55.7 ± 10.3	53.2 ± 13.5	*T* = 0.670; df = 38; *p* = 0.507
Sex (female/male)	4/16	5/15	χ^2^ = 0.143; df = 1; *p* = 0.705
Educational years	9.5 ± 1.7	10.8 ± 2.2	*T* = 2.011; df = 38; *p* = 0.051
Graduation (A levels, not)	6/14	6/14	n.a.
Handedness score*	84.0 ± 41.2	72.5 ± 46.4	*T* = 0.828; df = 38; *p* = 0.413
Mean hearing level (dB HL)	38.9 ± 14.7	41.5 ± 14.0	*T* = 0.560; df = 38; *p* = 0.579
Intelligence (MWT-B)	107.3 ± 12.1	116.7 ± 12.4	*T* = 2.439; df = 38; *p* = 0.020
Intelligence (ZVT)	88.6 ± 9.4	96.8 ± 12.2	*T* = 2.367; df = 38; *p* = 0.023
Hyperacusis (G	10.9 ± 9.6	3.6 ± 3.1	*T* = 3.250; df = 38; *p* = 0.002
Depressivity (MDI)	5.7 ± 5.7	2.9 ± 4.2	*T* = 1.784; df = 38; *p* = 0.082
Tinnitus duration (years)	12.7 ± 10.5	n.a.	n.a.
Tinnitus distress (TQ)	32.8 ± 20.1	n.a.	n.a.
Tinnitus loudness (0–10)	6.5 ± 1.7	n.a.	n.a.
Tinnitus laterality (left > right; right > left; left = right / within the head)	3/2/15	n.a.	n.a.
Interval between measurements (days)	7.7 ± 3.4	7.5 ± 1.3	*T* = 0.248; df = 38; *p* = 0.805
Motor threshold measurement 1	53.4 ± 4.3	56.1 ± 7.7	*T* = 1.784; df = 38; *p* = 0.190
Motor threshold measurement 2	54.9 ± 5.0	56.1 ± 6.7	*T* = 0.679; df = 38; *p* = 0.501

*Handedness with a scale ranging from −100 (left-handed) to +100 (right-handed).

### rTMS Protocol

Each subject was stimulated while wearing the EEG cap. Each stimulation protocol consisted of 200 pulses applied with 1 Hz at 60% stimulator output lasting about 3 min and 20 s. Stimulation intensity was set to a fixed value of 60% stimulator output and not adjusted to the individual motor threshold as stimulation was done through the EEG cap hampering exact motor threshold measurements. Nevertheless, motor threshold was determined in each participant by beginning the search for the hot spot in the area of C3 followed by stepwise adjustment of the stimulation intensity. Motor threshold was defined as the minimal stimulation intensity at which visible muscle twitches of the fingers of the right hand were obtained in at least five out of ten pulses. In most cases the motor threshold was ≤60% stimulator output with the exception of one patient in session one and another patient in session two. Three controls showed motor thresholds above 60% at session one and two. Motor thresholds did neither differ significantly between session 1 and 2 for both groups (patients: *T* = 1.974; df = 19; *p* = 0.063; controls: *T* = 0.046; df = 19; *p* = 0.964) nor between groups for session 1 and 2 (Table 1). After each stimulation protocol tinnitus patients were asked to rate the loudness change of their tinnitus on a percentage scale.

Pulses were delivered with a Medtronic system (Medtronic, USA) and a passive-cooled figure of eight coil (MCF B-65). For the temporal stimulation the coil was positioned over the left auditory cortex by using a standard procedure based on the 10-20-EEG system: T3/T4 served as starting point from which we measured 2.5 cm upwards following the line between T3/T4 and Cz. Then, we measured another 1.5 cm in the posterior direction perpendicular to the line T3/T4-Cz (Langguth et al., 2006). As a result the geometric center of the coil was located midway between C4/C5 and CP4/CP5 with the handle pointing upwards. For the prefrontal stimulation the geometric center of the coil was placed at F3/F4 with the handle pointing backwards with an angle of 45° to the sagittal midline. For the sham condition (single blinded; patients were not informed about a placebo stimulation) the back-side of the passive cooled coil was put over the electrode position CPz with the handle pointing backwards. On the back-side of the coil the magnetic field is reduced by a factor of six as shown by own measurements (for technical details; Van Doren et al., 2015). Vibration and click artifacts were well mimicked by this sham procedure.

### EEG Measurement

Five minutes of resting state EEGs were recorded before and after each rTMS protocol. Sixty two equidistant electrodes that were mounted in an elastic cap (EasyCap, Germany) were referenced to FCz during recording. Measurements were done with eyes closed. Impedances were kept below 10 kΩ. The signals were digitized at a rate of 500 Hz (BrainAmp DC, Vision Recorder, Brain Products, Germany).

### Data Analyses

After recording, EEG data were filtered with a high-pass filter of 1 Hz and a low-pass filter of 45 Hz and segmented into epochs of 2 s skipping the first and last two segments of the recording. Thereafter segments were visually inspected for distinct and visible aberrations from the overall recording to identify muscle artifacts (short high frequency oscillations), single channels with low signal-to-noise ratio (zero line in the EEG, main hums, or not smooth trajectories), or other large amplitude physiological artifacts (movement artifacts). Single segments were refused. The data excluding electrodes with low signal-to-noise ratio (maximum of five per measurement) was then subjected to an infomax independent component analysis in order to identify artifact components. Horizontal and vertical eye movement artifact components were removed and the remaining components were back-projected to the EEG signal space. Finally, the data was carefully visually inspected a second time for any remaining artifacts. Thereafter the data was re-referenced to an average reference, the online-reference FCz was reconstructed, and electrodes with signal loss were interpolated.

After preprocessing which was done with EEGLAB (Delorme and Makeig, 2004), data were converted into Fieldtrip format (Oostenveld et al., 2011) for power spectrum analysis. For power analyses we used the minimal number of available epochs of each measurement (all subjects and all conditions) and therefore chose the first 79 epochs of each measurement. After the fast fourier transformation using a hanning window (Fieldtrip parameters mtmfft and hanning) the power spectrum of each channel, condition, and subject was normalized by dividing the power of each frequency bin by the mean power of the whole power spectrum. The first EEG recording in session 1 and 2 served as baseline condition. For the baseline and sham conditions the two available measurements (one from session 1, one from session 2) were averaged.

We were interested in the rTMS induced changes between patients and controls. Thus, data of baseline measurements were subtracted from the post-stimulation conditions as indicators for rTMS induced changes (sham minus baseline, left-frontal minus baseline, etc.) (a comparable statistical approach can be found in Lorenz et al., 2010). In a second step, we substracted the baseline-corrected data of the sham condition from the baseline-corrected data for the different active conditions (stimulation site specific changes minus sham-induced change). These baseline- and sham-controlled data of patients and controls were compared in an unpaired *t*-test using a non-parametric permutation test (Fieldtrip parameter montecarlo) with 1000 iterations and a cluster correction to control for alpha inflation due to multiple testing of 63 electrodes. In other words we calculated the following *t*-test for each stimulation site: patients [(verum-baseline)-(sham-baseline)] vs. controls [(verum-baseline)-(sham-baseline)]. These contrasts were done to identify frequency-specific effects of the four stimulated cortical sites by repeating the *t*-tests for* a priori* defined frequency bands (delta: 2–3.5 Hz; theta: 4–7.5 Hz; alpha1: 8–10 Hz; alpha2: 10.5–12.5 Hz; beta1: 13–18 Hz; beta2: 18.5–21 Hz; beta3: 21.5–30 Hz; gamma: 30.5–44 Hz) as suggested by former studies (Vanneste et al., 2011b).

Averaged baseline-corrected data of significant clusters were exported into SPSS 22 (IBM Inc., USA) and were analyzed by 2 × 2 analyses of variance (ANOVAs) with the within-subjects factor stimulation protocol (verum rTMS intervention vs. sham) and the between-subjects factor group (patients vs. controls). This was repeated four times according to the four active stimulation sites. To control for group differences in hyperacusis, depressivity, years of education and intelligence these variables were used as covariates. Only effects which were significant for the ANOVA with and without covariates are reported here. In case of significant interaction effects in the ANOVA, *post hoc* Student *t*-tests were done. For the illustration of the results exact statistical values were obtained from the SPSS analyses and heat brain maps were generated by Fieldtrip using *t*-values of the group contrast for baseline- and sham-controlled data. If not otherwise specified default values for data pre-processing and analyses were used. As we were interested especially in rTMS induced changes in EEG power we did not present data with respect to group differences for baseline resting state EEG.

For reasons of completeness, baseline and baseline-corrected EEG power for the frequency bands and for both groups for the different stimulation conditions are presented in Supplementary Figures 1, 2.

## Results

### Changes in Tinnitus Loudness

In the first session four patients reported changes in tinnitus loudness only in verum conditions (one subject after left frontal stimulation with 30% reduction; one with 30% increase after left temporal stimulation; one with 60% reduction after right frontal stimulation; one subject with 15% increase after left frontal and right temporal stimulation). Three patients reported changes after verum and sham stimulation. Five of these eight patients also noticed changes in the second session. In the second session three out of the five patients reported changes only during the verum conditions again (one with 60% reduction after right frontal stimulation; one with 50% reduction after left temporal stimulation; and one with 20% increase after right temporal stimulation). Two patients reported changes after sham stimulation. Thus, in our sample only three patients reported reliably sham-controlled changes in tinnitus loudness after 1 Hz rTMS in different cortical stimulation sites. Due to the small number of patients with reliable change of tinnitus loudness after rTMS we abstained from correlating tinnitus-modulation and EEG power.

### Changes in Oscillatory Power

We were interested in the rTMS induced changes in oscillatory power (neuroplasticity) between patients and controls (Chung et al., 2015; Leuchter et al., 2015). Thus, we present significant group differences in neuroplasticity i.e., differences in post-stimulation activity controlled for pre-stimulation activity. These group differences are indicated by interaction effects of 2 × 2 ANOVAs with the factor group (patients vs. controls) and the factor stimulation protocol (verum vs. sham). As we conducted four active conditions (left and right temporal, left and right prefrontal) we run four ANOVAs. In the next step, significant effects were analyzed in a *post hoc* way by calculating contrasts between patients and controls and between verum and sham stimulation for the baseline-corrected activity (post- minus pre-stimulation). Please note that F-statistics indicate interaction effects (sham-controlled group differences in neuroplasticity) and T-statistics are indicating *post hoc* tests.

**Figure 2:**
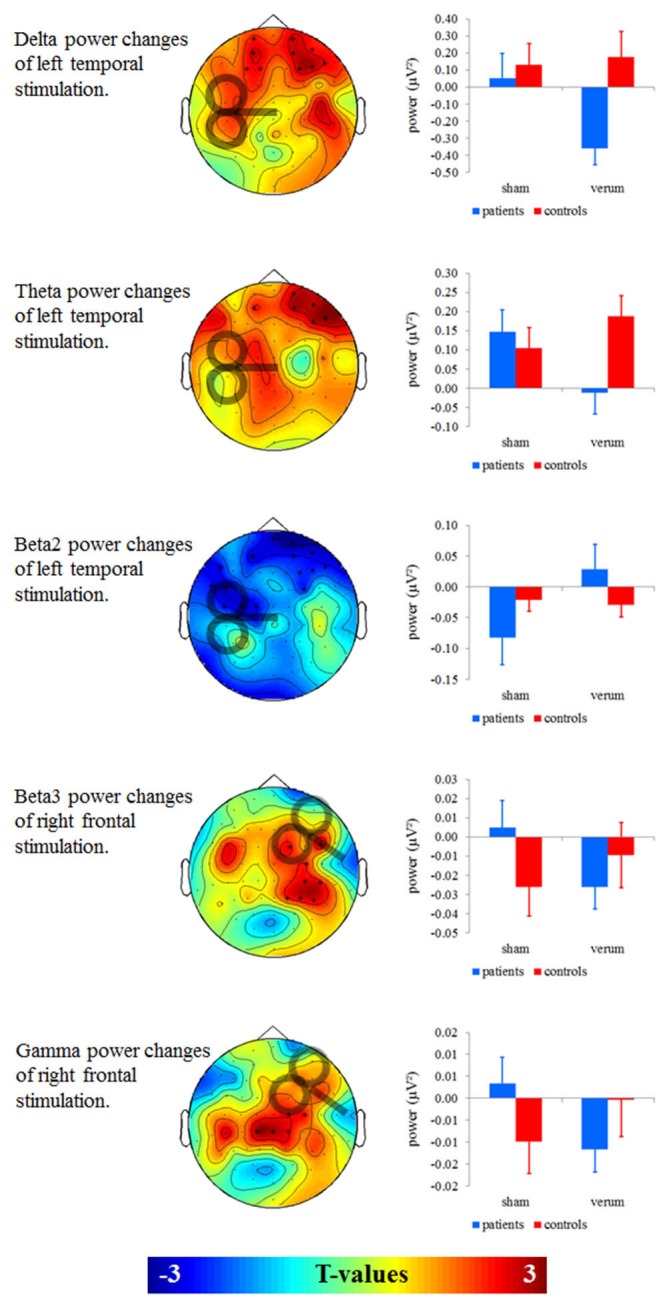
**Left**: T-maps for baseline- and sham-controlled group contrasts. Significant clusters of electrodes are marked with asterisks. **Right**: Baseline-corrected mean power of significant clusters was given in a corresponding interaction term with the factors group and stimulation (verum vs. sham).

We found no significant effects for left frontal and right temporal stimulation on oscillatory power (Figure 2). For left temporal stimulation, we found significant effects for the delta (*F* = 7.626; df = 1, 38 *p* = 0.009), theta (*F* = 9.781; df = 1, 38 *p* = 0.003) and beta2 power (*F* = 10.454; df = 1, 38 *p* = 0.003) in frontal electrodes. For delta and theta bands (Figure 2, two top panels) patients showed power decreases after verum as compared to sham stimulation (delta: *T* = 2.964; df = 19; *p* = 0.008; theta: *T* = 3.759; df = 19; *p* = 0.001) whereas controls showed no significant changes (delta: *T* = 0.495; df = 19; *p* = 0.626; theta: *T* = 1.275; df = 19; *p* = 0.218). Groups differed significantly after left temporal (delta: *T* = 2.840; df = 38; *p* = 0.007; theta: *T* = 2.588; df = 38; *p* = 0.014), but not after sham stimulation (delta: *T* = 0.452; df = 38; *p* = 0.654; theta: *T* = 0.543; df = 38; *p* = 0.590). For beta2 (Figure 2, middle panel) patients showed significant power increases after left temporal as compared to sham stimulation (*T* = 3.282; df = 19; *p* = 0.004). Controls showed no differences between verum and sham stimulation (*T* = 0.548; df = 19; *p* = 0.590). Groups did not differ after verum (*T* = 1.270; df = 38; *p* = 0.212) and sham stimulation (*T* = 1.313; df = 38; *p* = 0.197). To sum up the effects induced by left temporal stimulation, delta and theta power increased and beta2 power decreased in the group of patients.

For right prefrontal stimulation, we found significant effects for beta3 (*F* = 12.702; df = 1, 38; *p* = 0.001) and gamma power (*F* = 8.304; df = 1, 38; *p* = 0.006) in right temporal electrodes (Figure 2, two bottom panels). On a significant or near-significant level patients showed decreases in beta3 and gamma power after verum as compared to sham stimulation (beta3: *T* = 2.900; df = 19; *p* = 0.009; gamma: *T* = 2.046; df = 19; *p* = 0.055) whereas controls showed power increases (beta3: *T* = 2.072; df = 19; *p* = 0.052; gamma: *T* = 2.201; df = 19; *p* = 0.040). Groups did not differ significantly after the right prefrontal (beta3: *T* = 0.810; df = 38; *p* = 0.423; gamma: *T* = 1.150; df = 38; *p* = 0.257) or after the sham stimulation (beta3: *T* = 1.513; df = 38; *p* = 0.138; gamma: *T* = 1.398; df = 38; *p* = 0.170). To sum up the effects induced by right prefrontal stimulation, beta3 and gamma power decreased in the group of patients and increased in the group of controls.

## Discussion

### Changes in Tinnitus Loudness

In our sample of 20 patients with chronic tinnitus only three patients reported changes in tinnitus loudness after active 1 Hz rTMS in different cortical stimulation sites, but not after sham rTMS. These changes included both increases and decreases of tinnitus loudness. There were also four patients who reported changes after both verum and sham stimulation. The remaining 13 patients did not report any immediate effects. There are several reports that single sessions of rTMS can change the loudness of tinnitus (e.g., Plewnia et al., 2003; De Ridder et al., 2005; Müller et al., 2013). We chose 200 pulses and a frequency of 1 Hz as direct comparison with other protocols revealed that 1 Hz rTMS produces the most reliable changes in oscillatory brain activity (Müller et al., 2013). Moreover, several studies suggested that this protocol can reduce tinnitus loudness transiently in a subgroup of tinnitus patients (De Ridder et al., 2005; Meeus et al., 2009; Vanneste et al., 2011a). Several reasons may account for the small effects of 1 Hz rTMS in our study on tinnitus loudness. Firstly, effects of 1 Hz rTMS in earlier studies were rather small (De Ridder et al., 2007; Meeus et al., 2009; Müller et al., 2013) secondly rTMS effects of specific protocols depend on the tinnitus type (De Ridder et al., 2007; Meeus et al., 2009), and thirdly effects of 1 Hz rTMS in temporal cortex are related with acoustic stimulation before rTMS stimulation (Weisz et al., 2012). To conclude, the small effects on tinnitus perception in our study might be due to a complex interaction of rTMS protocol, tinnitus type, and ongoing temporal cortex activity. This finding is in line with a recent combined TMS-MEG study in ten patients with chronic tinnitus, in which different rTMS protocols (1 Hz, continuous and intermittent theta burst stimulation, and rTMS at the individual alpha peak frequency) showed only small and highly variable changes in tinnitus loudness (Müller et al., 2013).

We hypothesized that 1 Hz rTMS is able to induce tinnitus loudness reductions. Here we find sham-controlled tinnitus reduction in only a very small subsample of three subjects. This is in line with previous research concluding that rTMS is moderately effective in reductions of tinnitus loudness (Langguth et al., 2012; Lehner et al., 2012). We furthermore expected an association of tinnitus modulation with gamma power changes. However, this small sample of only three patients prevents a correlation between tinnitus-modulation and EEG power. We found changes in gamma power after right prefrontal rTMS. However, these and changes in other frequency bands after left temporal and right prefrontal 1 Hz might be rather unspecific and it remains unclear whether there is a relation to changes in tinnitus loudness.

### Changes in Oscillatory Power

After left temporal and right frontal stimulation we found significant power differences between tinnitus patients and controls on specific frequency bands. The observed changes (delta and theta decreases in frontal sensors and beta3 and gamma decreases in right temporal sensors) fit well with previous work which shows tinnitus-related changes in resting state EEG for these frequency bands (Vanneste and De Ridder, 2012). The most consistent finding from EEG and MEG studies seems to be increased gamma power, which has been found in tinnitus patients as compared to controls and which correlates with tinnitus distress, tinnitus intensity, or tinnitus duration (Vanneste and De Ridder, 2012). An increase of delta activity has been found in tinnitus patients in resting state MEG (Weisz et al., 2005; Adjamian et al., 2012). In a further EEG study delta and gamma power was positively correlated with tinnitus presence (Meyer et al., 2014). In further EEG studies, theta and also beta3 was shown to be associated with tinnitus or specific aspects of tinnitus (for an overview, see Vanneste and De Ridder, 2012).

Beside these cross-sectional and correlational approaches several interventional studies have shown that tinnitus reduction is related to decreases in delta/theta as well as in beta/gamma power. Tinnitus masking or residual inhibition decreases delta power (Kahlbrock and Weisz, 2008; Adjamian et al., 2012). Neurofeedback training with alpha/delta ratio from frontal electrodes as feedback signal leads to tinnitus loudness decreases (Dohrmann et al., 2007). Reduction of tinnitus severity using a specific form of auditory stimulation (coordinated reset) was shown to be related to a reduction of delta and gamma power in temporal, parietal, and cingulate regions (Tass et al., 2012; Adamchic et al., 2014).

Remarkably the present findings (decreases in delta, theta, beta3 and gamma band after 1 Hz rTMS left temporal or right frontal) resemble the findings in the mentioned studies, even if in the present study no relevant changes were detectable at a perceptual level. However, the tested intervention (1 Hz rTMS) has shown relevant clinical effects if applied with more stimuli and in repeated sessions (Soleimani et al., 2015), suggesting that the observed EEG changes may represent a more sensitive marker for the induction of therapeutic effects, than the patients’ report. However, we are well aware that at the current stage we cannot differentiate whether the observed rTMS induced changes of oscillatory brain activity are an indicator for a therapeutic effect or whether they just reflect an altered reactivity of tinnitus brains as a hint for metaplastic alterations in chronic tinnitus. Further studies will be needed to test whether EEG changes after single sessions may represent a valid predictor for long-term clinical effects of repeated sessions of rTMS treatment.

The observed effects in our study are compatible with the idea that tinnitus is related to thalamocortical dysrhythmia (Llinás et al., 1999; Adjamian et al., 2009; Adjamian, 2014; De Ridder et al., 2014, 2015) and that rTMS exerts its effects by attenuating thalamocortical dysrhythmia (Langguth et al., 2010). In short, the model of thalamocortical dysrhythmia postulates that the thalamus and cortex are interconnected with a rhythmic activity. Alpha activity is slowed down after deafferentiation (e.g., due to hearing loss) leading to increased delta and theta activity. This in turn leads to increased gamma activity in neighboring neurons based on mechanisms of lateral inhibition. Slow waves are considered to be markers of deafferentiation or neuroplastic processes (Adjamian et al., 2009; Assenza et al., 2015) whereas gamma is suggested to represent a neural correlate of the tinnitus percept/intensity (Vanneste and De Ridder, 2012).

In contrast to delta, theta, beta3, and gamma, beta2 power was increased in the patients after left temporal stimulation. The interpretation of this finding is ambiguous as changes in beta2 were only reported in two papers showing increased beta2 and beta3 for patients with chronic in contrast to recent onset tinnitus (Vanneste et al., 2011b) and increased alpha2, beta1, and beta2 in patients with high tinnitus distress (Joos et al., 2012). If the alpha and the lower two beta bands were interpreted as one entity, the finding would fit the thalamocortical dysrhythmia model which predicts decreased alpha activity (Weisz et al., 2005).

Focusing on neuroplastic changes in healthy controls our findings do not fit to previous effects of 1 Hz rTMS studies. Twelve healthy controls treated over the right dorsolateral prefrontal cortex with 1200 stimuli and 130% motor threshold showed increases in theta power in the contralateral prefrontal cortex within 60 min after the stimulation (Schutter et al., 2001). In another study, 27 healthy controls showed increases in alpha and beta power in occipital electrodes after right-temporal rTMS (Kim et al., 2012). These authors used 1800 pulses with 110% motor threshold. In addition, 20 healthy controls were stimulated at the left prefrontal cortex and showed decreases in all analyzed frequency bands (delta—gamma) with global effects in lower frequencies getting more focused to left prefrontal electrodes with higher frequencies (Woźniewska et al., 2013). These effects were induced by using 800 pulses at 120% motor threshold using breaks of 33 s after 200 pulses. In our healthy sample, we found significant increases in beta3 and gamma power in right temporal areas induced by right prefrontal stimulation using 200 pulses at 60% stimulator output.

As each of the four cited papers revealed different findings, this heterogeneity do not challenge the validity of the here reported findings. Heterogeneity might be related to methodological differences between studies (i.e., differences in number of applied TMS pulses, stimulation intensity). Otherwise variability in neuroplasticity as elicited with non-invasive brain stimulation is well known. Relevant factors are manifold (synaptic history, sample characteristics, etc.) and are focus of debate (Ridding and Ziemann, 2010).Thus, future work should include careful reports of relevant parameters of neuroplastic variability and should increase the homogeneity of these factors over different studies.

### Stimulation Site Specific Effects

Besides the frequency specific findings, also stimulation site specific effects were seen in the present work. Tinnitus patients showed changes in EEG power only for left temporal and right prefrontal stimulation. This finding confirms earlier MEG and EEG studies that have identified the right frontal and the left temporal areas as relevant hubs in tinnitus related neuronal network alterations.

In an MEG study the tinnitus related alpha network changes were most pronounced in the left temporal and right frontal cortex (Schlee et al., 2009). Moreover with increased tinnitus duration the connectivity between left temporal and right frontal cortex further increases (Schlee et al., 2009; Vanneste et al., 2011b). In another EEG study gamma band activity in the left temporal and right frontal cortex was related to pitch change after coordinated reset therapy (Adamchic et al., 2012).

Low frequency rTMS for tinnitus treatment has mostly been performed over the left temporal cortex (Lefaucheur et al., 2014) and in one study also over the right dorsolateral prefrontal cortex (Kreuzer et al., 2011). Early rTMS studies in chronic tinnitus assumed that the main target for treatment of tinnitus might be the left temporal cortex (Eichhammer et al., 2003). This assumption was based on PET studies that showed hyperactivity of the left temporal cortex irrespective of tinnitus laterality (Arnold et al., 1996; Langguth et al., 2006; Schecklmann et al., 2013). However, this concept has been questioned recently as it was demonstrated that left-sided hyperactivity is independent from tinnitus as healthy controls show the same pattern (Geven et al., 2014) and as rTMS of the temporoparietal cortex contralateral to the tinnitus perceipt was shown to be more effective (Khedr et al., 2010a).

The finding that 1 Hz stimulation shows only effects if applied over the right (in contrast to the left) prefrontal cortex fit well to established rTMS protocols for the treatment of affective disorders. Based on a hemispheric dysbalance model which assumes decreased left-frontal and increased right-frontal activity in affective disorders (Grimm et al., 2008; Vanderhasselt and De Raedt, 2009) rTMS for affective disorders is either performed as left-frontal high-frequency rTMS or as right-frontal low-frequency stimulation (Lefaucheur et al., 2014). Similar protocols in combination with left temporal stimulation (Kleinjung et al., 2008; Kreuzer et al., 2011; Lehner et al., 2013; Langguth et al., 2014) have also shown moderate efficiency in tinnitus patients.

### Conclusion

As hypothesized we could demonstrate that neuroplastic effects after rTMS interventions over temporal and frontal brain areas differed between tinnitus patients and controls with effects taking place in other areas of the brain than the stimulated region. Tinnitus loudness reductions were scarce and could therefore not be linked to changes in gamma power. The present data show that combined TMS-EEG measurements can serve as complementary and supplemental neuroscientific measurement to already existing techniques such as resting state and sound-evoked fMRI or EEG/MEG. Here we could demonstrate that the effects of low frequency rTMS are specific for stimulation site and EEG frequency band and differ between tinnitus patients and healthy control subjects. This indicates that this method has potential for detecting tinnitus-related changes in neuroplasticity, i.e., changes in resting state activity after rTMS. The observed effects can be interpreted within the thalamocortical dysrhythmia model assuming that slow waves represent processes of deafferentiation and that high frequencies might be indicators for tinnitus loudness.

Earlier MEG and EEG studies have identified the left temporal and the right frontal areas as relevant hubs in tinnitus related neuronal network alterations with involvement of non-auditory areas in tinnitus distress. In line with those findings main treatment targets of 1 Hz rTMS were the left temporal cortex in chronic tinnitus and the right frontal cortex in depression. The aim of future studies should be the evaluation of the effects of different rTMS protocols on tinnitus loudness and oscillatory power. There is good evidence that daily sessions of non-invasive brain stimulation are superior to single sessions (Vanneste and De Ridder, 2013; Claes et al., 2014) and that effects of single sessions can guide the selection of rTMS protocols for daily treatment (De Ridder et al., 2013).

## Author Contributions

MS, BL, JG, ES contributed to the conception or design of the study. MS, JG, and ES were involved in the acquisition, analyses, and interpretation of the results. MS, AL, WS, and BL were involved in the analyses and interpretation of the results. MS, AL, WS, and BL drafted the manuscript and revised it critically for important intellectual content. All authors gave final approval of the version to be published and agree to be accountable for all aspects of the work in ensuring that questions related to the accuracy or integrity of any part of the work are appropriately investigated and resolved.

## Conflict of Interest Statement

The authors declare that the research was conducted in the absence of any commercial or financial relationships that could be construed as a potential conflict of interest.

## Supplementary Material

Supplementary Figure 1 **EEG power (μV^2^) for the group of healthy controls**.

Supplementary Figure 2 **EEG power (μV^2^) for the group of patients with chronic tinnitus**.
