# Supplementary figures and images for: Repetitive transcranial magnetic stimulation induces oscillatory power changes in chronic tinnitus
Source: Front Cell Neurosci. 2015 Oct 21;9:421. doi: 10.3389/fncel.2015.00421 (PMC4617176; doi:10.3389/fncel.2015.00421)

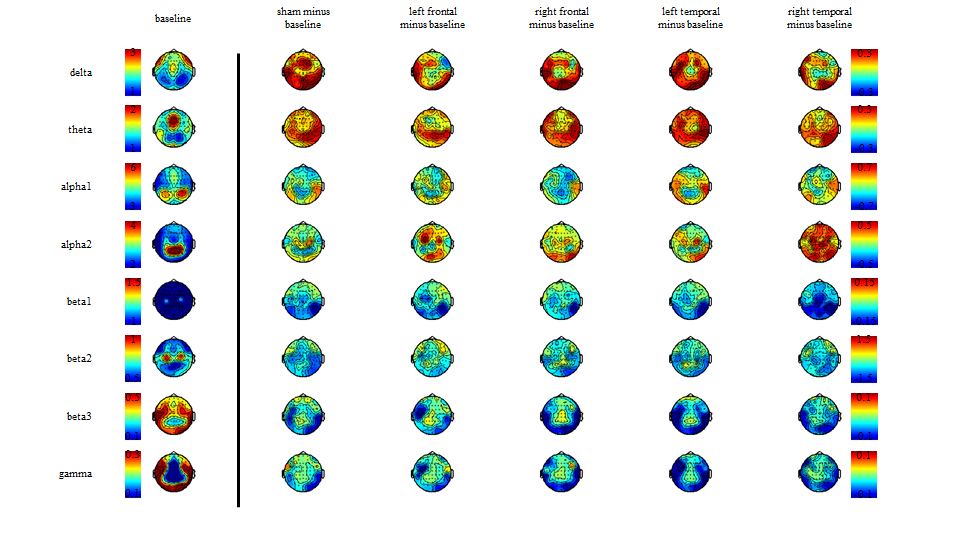

Supplement: Supplementary Figure 1 — EEG power (μV2) for the group of healthy controls. [file Image_1.tif]

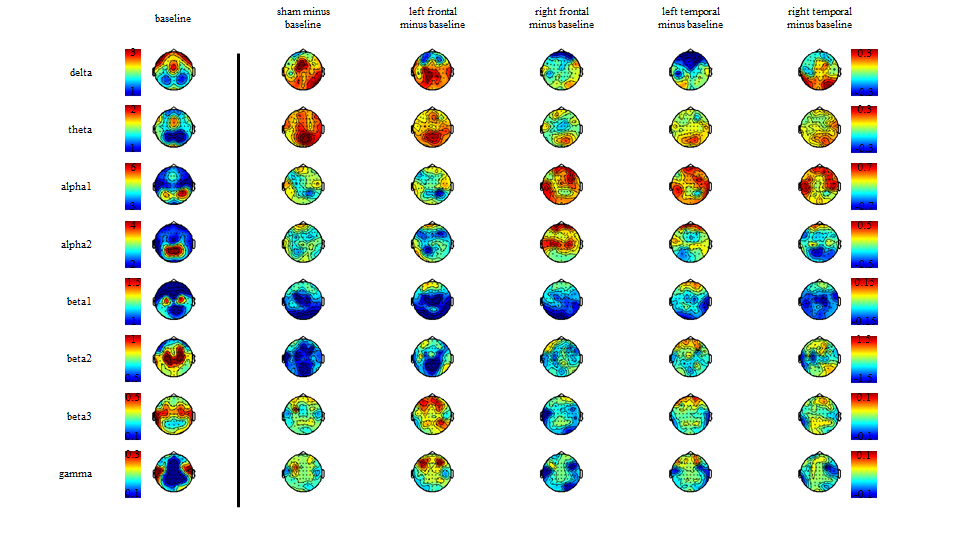

Supplement: Supplementary Figure 2 — EEG power (μV2) for the group of patients with chronic tinnitus. [file Image_2.tif]
